# Development of α-Tocopherol Succinate-Based Nanostructured Lipid Carriers for Delivery of Paclitaxel

**DOI:** 10.3390/pharmaceutics14051034

**Published:** 2022-05-11

**Authors:** Sushrut Marathe, Gauri Shadambikar, Tabish Mehraj, Suresh P. Sulochana, Narendar Dudhipala, Soumyajit Majumdar

**Affiliations:** 1 Department of Pharmaceutics and Drug Delivery, University of Mississippi, Oxford, MS 38677, USA; spmarath@go.olemiss.edu (S.M.); gdshadam@go.olemiss.edu (G.S.); tmehraj@go.olemiss.edu (T.M.); spsuloch@olemiss.edu (S.P.S.); ndudhipa@olemiss.edu (N.D.); 2 Research Institute of Pharmaceutical Sciences, University of Mississippi, Oxford, MS 38677, USA

**Keywords:** paclitaxel, α-tocopherol succinate, retinoblastoma, NLC, ocular drug delivery

## Abstract

The management of retinoblastoma (RB) involves the use of invasive treatment regimens. Paclitaxel (PTX), an effective antineoplastic compound used in the treatment of a wide range of malignant tumors, poses treatment challenges due to systemic toxicity, rapid elimination, and development of resistance. The goal of this work was to develop PTX-loaded, α-tocopherol succinate (αTS)-based, nanostructured lipid carrier (NLCs; αTS-PTX-NLC) and PEGylated αTS-PTX-NLC (αTS-PTX-PEG-NLC) to improve ocular bioavailability. The hot homogenization method was used to prepare the NLCs, and repeated measures ANOVA analysis was used for formulation optimization. αTS-PTX-NLC and αTS-PTX-PEG-NLC had a mean particle size, polydispersity index and zeta potential of 186.2 ± 3.9 nm, 0.17 ± 0.03, −33.2 ± 1.3 mV and 96.2 ± 3.9 nm, 0.27 ± 0.03, −39.15 ± 3.2 mV, respectively. The assay and entrapment efficiency of both formulations was >95.0%. The NLC exhibited a spherical shape, as seen from TEM images. Sterilized (autoclaved) formulations were stable for up to 60 days (last time point checked) under refrigerated conditions. PTX-NLC formulations exhibited an initial burst release and 40% drug release, overall, in 48 h. The formulations exhibited desirable physicochemical properties and could lead to an effective therapeutic option in the management of RB.

## 1. Introduction

Retinoblastoma (RB) is the most common intraocular cancer of childhood arising due to the development of the mutation or the loss of one allele for the RB1 gene [1,2]. Losing one allele can predispose the individual toward the development of the cancer, while losing both alleles leads to an initiation of the RB [3]. The incidence of RB is constant worldwide at one case per 15,000–20,000 livebirths, which corresponds to about 9000 new cases every year [2]. The disorder has no validated geographic or population hotspots. The greatest disease burden is recorded in large populations that have high birth rates, such as in Asia and Africa. The highest mortality of around 40–70% in children is found in these areas when compared with Europe, Canada, and the USA, which have mortality rates of 3–5% [1,2].

Paclitaxel (PTX), the most widely used anticancer drug, is applied for the treatment of various types of malignant diseases [4,5]. PTX induces cell apoptosis by binding and stabilizing the microtubules, thereby inducing the polymerization. This leads to the reduction in the amount of the tubulins in the cell, resulting in programmed cell death [4]. The RB cell lines are sensitive to low doses of PTX, and the drug has been shown to exert the cytotoxic effect at nanomolar concentrations [4].

Vitamin E, relatively non-toxic and well tolerated by humans, consists of groups of tocopherols (α, β, γ, δ) and tocotrienols (α, β, γ, δ) and are potent antioxidants because of their free phenol hydroxyl group [6]. Many published studies indicate that vitamin E is a candidate for the treatment of cancer as it is an important micronutrient that balances antioxidant and pro-oxidant reactions in the body [6,7]. α-Tocopherol succinate (αTS) has been reported to be effective in enhancing the tumor growth inhibition activity of anticancer agents [8]. αTS has been shown to inhibit tumor cell proliferation by mechanisms such as inhibiting DNA synthesis, constraining the cellular proteins necessary for regular cell cycle and by slowing down the cell cycle [9,10,11,12]. αTS as an active isomer of vitamin E is known for its potent antioxidant property. It suppresses the toxic effect of free radicals in the cells and prevents the inhibition or alteration of cell function caused by lipid peroxidation [8,12]. αTS has also been reported to induce apoptosis in erythroid leukemia and in prostate and breast cancer cells [6,13]. The literature reports show that αTS can successfully inhibit multiple types of cancer cells and can potentiate the effectiveness of the anticancer agents such as PTX [14,15], doxorubicin [13,16] and camptothecin [17] when used in combination [13,17,18].

Although PTX is a very potent anti-cancer agent, its use is challenged by its poor aqueous solubility and the need for solubilizers such as Cremophor^®^ EL and ethanol in high concentrations [19,20]. These limitations of the PTX can be overcome by lipid nanoparticle delivery systems that can have high drug loading and can carry the drug to the site of action, thus increasing the efficacy and bioavailability of PTX to the tumor cells [21,22,23]. The lipid matrix of the nanostructured lipid carriers (NLCs) helps to solubilize and entrap the hydrophobic compounds within themselves and prevents the precipitation of the drug in the dispersion media [24]. As per the biopharmaceutical classification system (BCS), PTX sits in the B8CS IV category because of its poor solubility and poor permeability [25]. Thus, for such a drug, it is necessary to have a carrier that can get the drug to the site of action and within the cells. Thus, for the efficient delivery of a highly hydrophobic compound such as PTX, with controlled release and long-term stability, NLC was selected as a primary means for delivery.

There has been a great focus on the biodegradable submicron-sized NLC with different lipid-based formulations of PTX, in the size range of 50–1000 nm, without and with different surface modifications. In recent years, their advantages such as the controlled release of the drug, protection of the drug against degradation, drug targeting, and safe biocompatible carrier have been outlined [21,22,23,26,27].

The combined delivery of PTX with αTS could potentiate the cytotoxic activity of the PTX by achieving synergistic effect. Furthermore, the combination of PTX with αTS showed significantly higher apoptotic activity on multidrug resistant phenotype cancer [28,29,30,31].

Polyethylene glycol (PEG) is a nonionic, hydrophilic polyether compound and has been demonstrated to be safe. PEG has been investigated for alteration of the surface of nanocarrier systems to modify their physiochemical characteristics. This process is called PEGylation, and it can provide various benefits such as increased static stability by forming a hydrophilic shell around NLC and increased circulation time by preventing uptake by macrophage (phagocytosis) opsonization [32]. For ocular drug delivery, PEG with molecular weights in the 2000 to 5000 Dalton range can provide favorable ocular permeation of molecules and also provide stability during autoclaving [33].

The objective of the current study was to encapsulate PTX in an αTS matrix and formulate stable long-acting NLC (αTS-PTX-NLC) for the treatment of RB. For the development of the αTS-PTX-NLC, repeated measures ANOVA analysis was used to analyze the full factorial design and to evaluate the effect of the formulation composition on the physical characteristics and stability of the NLC. The formulation composition with the least particle size and polydispersity index (PDI) that exhibited the minimum change over the period of study was selected for further analysis. The optimized formulation was then further evaluated with different levels of *N*-(carbonyl-methoxypolyethyleneglycol-2000)-1,2-distearoyl-sn-glycero-3-phosphoethanolamine sodium salt (DSPE-PEG-2k; αTS-PTX-PEG-NLC) to understand the effect of PEGylation on the physical characteristics of NLC.

## 2. Materials and Methods

### 2.1. Materials

PTX (99.5%), Lot no. P05F047 was purchased from Alfa AESAR, Haverhill, MA, αTS (RRR-α-Tocopheryl acid succinate) was purchased from Spectrum^®^ Chemical Mfg Corp., New Bruinswick, NJ, USA, Lot no. 2HE0577; Propylene glycol monocaprylate (Capryol™ 90) was a gift sample given by Gattefossé, France, Batch number 16093. *N*-(carbonyl-methoxypolyethyleneglycol-2000)-1,2-distearoyl-sn-glycero-3-phosphoethanolamine sodium salt (DSPE-PEG-2k) was purchased from Lipoid GmbH (Ludwigshafen, Germany). All other reagents were of high-performance liquid chromatography (HPLC) analytical grade.

### 2.2. Methods

#### 2.2.1. Analytical Method for PTX

The PTX was analyzed by modifying the HPLC method reported in the literature [34,35]. The HPLC system comprised a Waters Alliance e2695 separations module and Waters 2489 UV/Vis dual absorbance detector. A C_18_ column (4.6 × 250 mm, 5 µm particle size), λ = 227 nm, mobile phase consisting of acetonitrile and water in a 60:40 ratio, with a flow rate of 1.2 mL/min was used [34,36]. The samples were analyzed through Empower software, and the standard plot (0.1–20 µg/mL) was constructed using linear regression by plotting the AUC against the concentrations. The equation for the line and fit of the linear model (R^2^) was calculated. The assay, entrapment efficiency and release study samples were determined from the standard plot.

#### 2.2.2. Defining the Quality Target Product Profile (QTPP) and Critical Quality Attributes (CQAs)

The definite quality characteristics of the αTS-PTX-NLC was defined using QTPP to achieve the optimal NLC formulation for ocular delivery of the PTX. Various elements of QTPP for development of the αTS-PTX-NLC and αTS-PTX-PEG-NLC have been summarized in Table 1, while Table 2 enlists the respective justification of selecting each CQA.

#### 2.2.3. Initial Risk Assessment and Factor Screening Study

**Figure 1:**
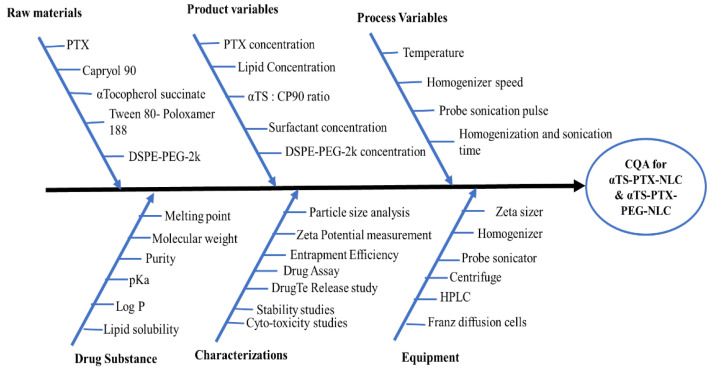
Ishiwaka diagram for the development of αTS-PTX-NLC and αTS-PTX-PEG-NLC formulations.

To identify the critical factors affecting the critical material attributes (CMAs) and/or critical process parameters (CPPs) for αTS-PTX-NLC and αTS-PTX-PEG-NLC affecting the CQAs of the drug product, an Ishikawa diagram was constructed to identify an initial list of potential high-risk factors that influenced the quality of the PTX-loaded αTS based NLC (Figure 1). Preliminary studies were carried out to find out CMAs/CPPs with high risk.

#### 2.2.4. Preparation of αTS-PTX-NLC and αTS-PTX-PEG-NLC

For the preparation of the NLC, liquid lipid was selected on the basis of the previously reported PTX nanolipid formulation [22]. PTX exhibited the highest solubility in Capryol™ 90 (CP90), and hence, it was selected as a liquid lipid for the development of the NLC [22].

The selected solid lipid, i.e., αTS, liquid lipid, i.e., CP90, and PTX were mixed, and this mixture was melted at 80 °C. Once the drug (0.1% *w*/*v*) was completely solubilized in the molten lipid mass, the heated aqueous phase containing 2.0% *w*/*v* of Tween^®^80, 0.5% *w*/*v* of Poloxamer^®^ 188 and 2.25% *w*/*v* of glycerin was added dropwise with stirring maintained at 2000 rpm to form a hot pre-emulsion. This pre-emulsion was homogenized with a T25 digital Ultra-Turrax (IKA, Staufen, Germany) homogenizer for 5 min at 15000 rpm, which was followed by probe sonication (SONICS^®^ Vibra-Cell™ 500 watts, Newtown, CT, USA) with a 3 mm stepped microtip probe for 10 min at 120 Hz (40% power and 115 V) with the pulse/frequency of 10 s On and 5 s Off. The prepared formulations will be stored at 4 °C. To modify the NLC surface with PEG and for the preparation of αTS-PTX-PEG-NLC (also known as PEGylation), DSPE-PEG-2k was added to the lipid mixture during the melting process.

#### 2.2.5. Particle Size Analysis, Poly Dispersity Index (PDI) and Zeta Potential (ZP) Determination

The physical characterization of the PTX-NLC was evaluated by determining the particle size, PDI and ZP using Zetasizer (Zetasizer Nano ZS Zen3600, Malvern Instruments, Inc., Malvern, PA, USA). The formulation was diluted 100 times with distilled water. Each sample was measured three times at 25 ± 0.1 °C.

#### 2.2.6. Entrapment Efficiency

To determine the entrapment efficiency (EE%) of the nanoparticles, 400 µL of the NLC formulation was added into the 10 kDa Amicon^®^ ultrafiltration vials and centrifuged for 15 min at 3000 rpm. The filtrate was analyzed with HPLC to determine the drug concentration. The entrapment efficiency of the nanoparticles was determined with the following equation:

(1) $$\mathrm{EE}\%=\frac{\mathrm{Amount}\;\mathrm{of}\;\mathrm{drug}\;\mathrm{in}\;\mathrm{formulation}-\mathrm{Amount}\;\mathrm{of}\;\mathrm{drug}\;\mathrm{in}\;\mathrm{the}\;\mathrm{filtrate}}{\mathrm{Amount}\;\mathrm{of}\;\mathrm{the}\;\mathrm{drug}\;\mathrm{in}\;\mathrm{formulation}}\times 100$$

##### Assay of PTX-Loaded NLCs

For the determination of PTX assay in the αTS-PTX-NLC formulations, 50 µL of the formulation was diluted to 5 mL in a volumetric flask with acetonitrile. This solution was sonicated for 10 min and centrifuged at 3000 rpm for 10 min. The supernatant was analyzed with HPLC.

#### 2.2.7. Sterilization of the Formulation by Autoclave

The formulations were sterilized by means of autoclaving using a Tuttnauer 3850EL sterilizer autoclave. The standard cycle with a temperature of 121 °C and pressure of 1 atm with the holding time of 15 min was utilized. The samples were analyzed to investigate the effect of sterilization on the physiochemical properties of the NLC formulations.

#### 2.2.8. In Vitro Drug Release

The release of the PTX from αTS-PTX-NLC and αTS-PTX-PEG-NLC was performed on day 1 as well as day 30 using a dialysis bag (MW 10 kDa), with release media composed of 5% methyl β-Cyclodextrin and 2% Tween© 80 and with 1 M sodium salicylate in phosphate buffer saline pH 7.4. Then, 2 mL of NLC formulation was added in the dialysis bag that was suspended in the 20 mL of release media. PTX solution (1 mg/mL) in DMSO was used as control. The study was performed at 35 °C and a stirring rate of 400 rpm. About 500 µL was withdrawn at each time interval, and an equivalent amount of fresh release media was added to maintain sink condition. The amount of the PTX in the samples was analyzed using HPLC. The experiment was performed in triplicate. The assessment of the release of the drug from the initial sample and stability sample was performed by calculating a similarity factor (ƒ_2_).

The following equation is used to calculate the similarity factor

(2) f2=50×log{[1+(1n)∑t=1n(Rt−Tt)2]−0.5×100}

where *R_t_* and *T_t_* are the percentage dissolved of the initial sample and stability sample, respectively, at each time point and *n* = number of time points. The two release profiles are considered similar if the obtained ƒ_2_ value is between 50 and 100. The *f*_2_ value less than 50 indicates dissimilarity between the two release profiles.

Along with the similarity factor, other models to understand the kinetics of the drug release were applied.

#### 2.2.9. Morphology of the PTX-NLC by Transmission Electron Microscopy (TEM)

The shape and structural morphology of the nanoparticles was deduced using TEM by negative staining protocol as follows. The grid was held over the 20 μL of sample for about 1 min followed by removal of the excess sample with the filter paper. The grid was washed by dipping in distilled water, and the excess was also removed using a filter paper. This grid was stained immediately using UranyLess contrast solution for 1 min followed by drying. This stained and dried grid was imaged by JEOL JEM-1400 Flash TEM and captured on a Gatan One View digital camera (studies performed at the University of Tennessee, Knoxville, TN, USA).

## 3. Results and Discussion

### 3.1. Optimization of the Lipid Ratio

The nanoparticle formulations with varying solid lipid and liquid lipid ratios were prepared and characterized for particle size, PDI, ZP, EE and assay for a stability of up to 30 days at 4 °C.

For the development of the NLC formulations, the selection of the solid lipid and liquid lipid plays a primary role. The essential properties for selection are solubility of the drug in the lipids and the miscibility of lipids with each other. α-Tocopherol is known to be a good solubilizer for hydrophobic drugs and is a good lipophilic compound; hence, it was used as a solid lipid in the lipid matrix [37,38]. Capryol™ 90 was selected as the liquid lipid from the solubility as reported in the literature [22]. The surfactants used in the formulation development were Tween^®^80 and Poloxamer^®^ 188, both of which are FDA-approved non-ionic surfactant and have been proven to be safe for ocular drug delivery [39,40]. The effects of homogenizer speed and duration have a significant effect on the particle size distribution of the NLC, and a speed of 15,000 rpm has been shown to be optimum to reduce the particle size while avoiding the charring of the lipids in the formulation [41].

The QTPP for the NLC formulations was selected based on reports in the literature [42,43]. The optimal particle size for nanoparticles for ocular drug delivery is between 50 and 400 nm, as these small particles have the ability to overcome physiological barriers such as corneal barrier and to carry the drug to cells or the intracellular spaces [44]. For the purpose of lipid-based nanocarrier systems, the PDI of 0.3 and below is considered to be acceptable, indicating a homogenous particle size distribution of the NLC [45]. The ZP of the NLC plays a significant role in the stability of NLC, preventing aggregation due to electrostatic repulsion. The NLC formulations with zeta potential values less than −30 mV or greater than +30 mV generally exhibit excellent colloidal behavior and repulsive forces strong enough to maintain stability. The formulations with low ZP will show instability due to aggregation caused by Van Der Waal inter-particle attractions [46,47]. To understand the effect of the amount of the lipids and the ratio of the lipids in the formulation, a study design as shown in Table 3 was performed.

The particle size is an important parameter for the ocular drug delivery. A high specific contact area can be achieved because of the smaller particle size of the nanoparticles, thus enhancing the drug delivery as well as higher drug permeation. Table 3 presents the particle size, PDI, and ZP of the nine formulations on day 1 and day 30.

The particle size and the PDI of the NLC formulations showed the variance over time and need to be stable on storage. Optimizing the formulation by traditional methods lacks requiring analysis of the data acquired at a single time-point. The trend in the change of characteristics of the samples over time cannot be tracked and utilized. Hence, to understand the effect of the storage stability on the characteristics of NLC by varying the lipid content and the lipid ratio, a two-way repeated ANOVA study design was utilized. The repeated measures ANOVA (RM ANOVA) has a primary advantage that it can be used to track a specific variable over time and understand the effect of another variable. This serves a unique advantage over ANOVA [48]. It has more statistical power, and in this RM ANOVA type of design, each subject in the study functions as an experimental block. A block can be defined as a categorical variable that can explain the variation in the dependent variable that arises independent of the factor that we want to see the effect from. Thus, the blocks in the experimental design can reduce the bias and variance of the error because of these nuisance factors [48,49].

A two-way repeated measures ANOVA was conducted on particle size, PDI, and αTS-PTX-NLC to evaluate the effects of the amount of solid lipid (*αTS*) and ratio of liquid lipid to the solid lipid (*CP90:αTS*) on storage stability (*stability*). Owing to the balanced nature of the study design, the normality and sphericity assumptions were met. In the repeated measures ANOVA, the effects exhibited by the independent variables on the same sample that is measured at different time points are called the within-subject effects (Table 4). These effects are expressed as the interaction between the independent variables and the time-dependent variable, in this case, storage stability (*stability*). Therefore, in the analysis, the effect of the concentration of the α-tocopherol succinate (*αTS*), and the ratio of the liquid lipid to solid lipid (*CP90:αTS*) on the particle size and PDI on storage is described as the interaction between storage *stability* and concentration of α-tocopherol succinate (stability*αTS), storage *stability* and the ratio of the liquid lipid to solid lipid (stability*CP90:αTS), and storage, concentration of α-tocopherol succinate and the ratio of the liquid lipid to solid lipid (stability*αTS*CP90:αTS).

The effect of the independent variables at different levels on the different samples can be explained by the between-subject effects (Table 5). They can be used to understand the direct effect of concentration of α-tocopherol succinate (*αTS*) and ratio of liquid lipid to solid lipid (*CP90:αTS*) on particle size and PDI of nanoparticles.

**Figure 2:**
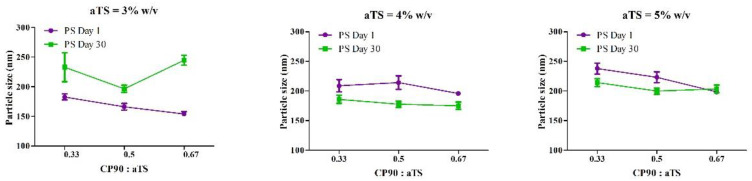
Descriptive plots for the effect of the amount of αTS and CP90:αTS ratio on the particle size when the concentration of αTS is 3%, 4% and 5%.

**Figure 3:**
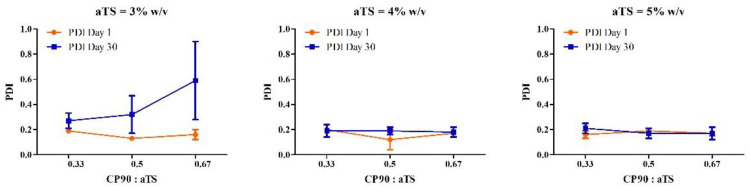
Descriptive plots for the effect of the amount of αTS and CP90:αTS ratio on the PDI when the concentration of αTS is 3%, 4% and 5%.

The within-subject interactions of the stability*αTS and stability*CP90:αTS on the particle size of NLC were significant (*p* < 0.001), indicating a significant change in the particle size measured on day 1 and day 30 of the formulations with different compositions, i.e., with concentrations of α-tocopherol succinate and Capryol™ 90: α-tocopherol succinate ratios. From Figure 2, the formulation with 4% αTS showed the least change in particle size distribution on storage. For the PDI of each individual formulation between day 1 and day 30, the three-way interaction stability*αTS*CP90:αTS was significant (*p* = 0.029), indicating a significant effect of all the variables (*concentration of αTS*, *the ratio of the liquid lipid and solid lipid*, and *storage stability*) together on the changes in PDI observed between day 1 and day 30 (Figure 3).

The between-subject effects from the analysis indicated that there was a significant difference between the particle size of the NLC formulations by varying the concentration of the α-tocopherol succinate (*αTS*) and the lipid ratios (*CP90:αTS*) as evidenced by the significant interaction αTS*CP90:αTS (*p* = 0.02). While in the case of the PDI, only the concentration of the α-tocopherol succinate (*αTS*) had a significant effect, indicating that by changing the concentration of α-tocopherol succinate, the PDI can be moderated. The ratio of the liquid lipid to solid (*CP90:αTS*) did not have any significant effect on the difference of PDI between the different formulations on its own (*p* = 0.275) or as an interaction with the concentration of α-tocopherol succinate (*p* = 0.233).

**Figure 4:**
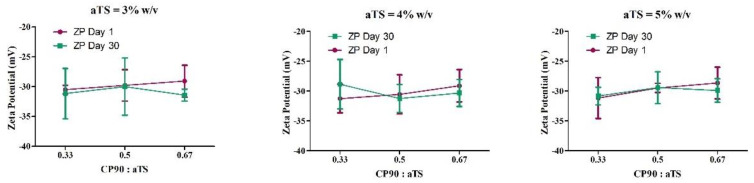
Descriptive plots for the effect of the amount of αTS and CP90:αTS ratio on the zeta potential when the concentration of αTS is 3%, 4% and 5%.

In case of the ZP (Figure 4), for the within-subject effect as well as between-subject effect, no significant change was observed for the NLC formulations. The nanoparticles with zeta potential greater than +30 mV or less than -30 mV are considered strongly cationic or strongly anionic, respectively [50]. This strong charge on the nanoparticles not only provides colloidal stability by means of electrostatic repulsive forces but also is critical to achieve higher membrane permeation for drug delivery [50,51]. All the formulations exhibited a zeta potential close to, or less than, −30 mV both on day 1 and day 30 of studies. Hence, zeta potential was not considered as a significant characteristic to evaluate and optimize the formulations.

Post hoc analysis was performed to understand the main effects of the independent variables as well as the interactions. The formulations F5 and F6 exhibited the best characteristics amongst all formulations tested, and it was observed that there was no statistical difference between the formulation F5 and F6 for particle size, PDI and zeta potential—*p* > 0.05. Furthermore, there was no significant difference in the composition (except CP90:αTS ratio 0.5 and 0.67% for F5 and F6, respectively). Hence, we selected only one formulation (F5) for further studies.

Because of the low solubility of PTX, the drug concentration in the formulation was found to be a critical parameter for the physical stability of NLC, and higher concentrations of PTX induced precipitation during the preparation of formulations. The drug loading at 0.1% *w*/*w* was selected as optimum drug loading based on preliminary screening studies.

The drug loading for the optimized formulation was found to be 1.5–10 times the drug loading, as reported by the previously developed formulations [22,52,53].

### 3.2. Optimization of DSPE-PEG-2k Concentration

The formulation F5 was selected from the previously mentioned studies and different concentrations of DSPE-PEG-2k were tried to understand the effect of PEGylation on the physiochemical characteristics of the NLC (Table 6). The placebo formulations were also included to understand the effect of the drug loading on PEGylation.

**Figure 5:**
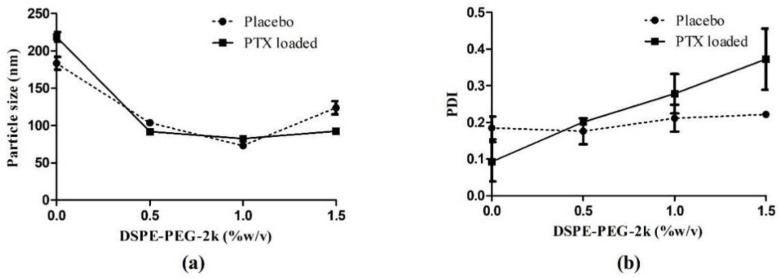
Effect of the concentration of DSPE-PEG-2k on the particle size (**a**) and PDI (**b**) of the NLC formulations.

From the above results (Figure 5 and Table 7), there is a significant reduction in particle size of the NLC on PEGylation (*p* < 0.001). This can be attributed to the amphiphilic nature of the DSPE-PEG-2k molecule, where the lipophilic DSPE end stays with the lipids and PEG protrudes out of the NLC [54], thus helping stabilize the NLC at a much lower particle size. The interaction of the DSPE-PEG2k*PTX was significant (*p* < 0.001), indicating that there is a significant difference in the particle size of the placebo and the drug-loaded formulations, and they showed different trends with increasing the DSPE-PEG-2k in the formulation.

In case of the PDI of the formulations, the placebo formulations showed a non-significant increase with increasing DSPE-PEG-2k concentrations. While in case of the drug-loaded formulations, there was an almost linear increase with the increasing concentrations of DSPE-PEG-2k.

From the above, the 1% concentration of the DSPE-PEG-2k was selected for further studies.

### 3.3. Drug Release Study

**Figure 6:**
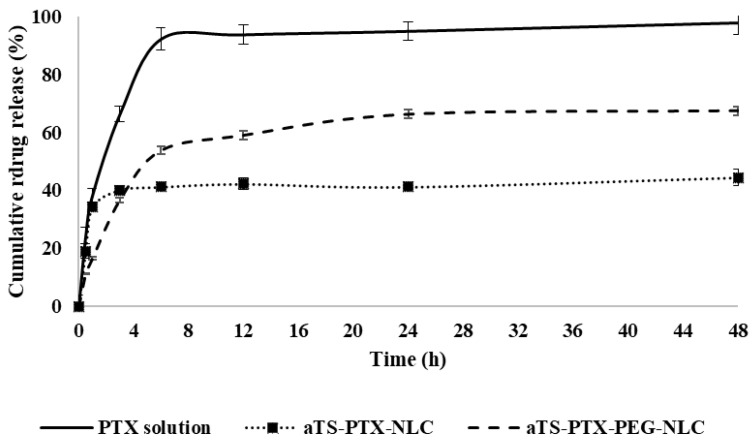
Release of PTX from αTS-PTX-NLC, αTS-PTX-PEG-NLC and control solution.

The drug release study was carried out in release media containing Tween 80 and Cyclodextrin along with sodium salicylate. Here, the sodium salicylate acts as a hydrotropic agent, enhancing the solubility of the drug in water [55]. The αTS-PTX-NLC showed controlled release of the drug from the formulation when compared against the PTX solution. The PTX solution used as control exhibited rapid drug release, and about 100% of the drug was found in release media in 4 h. As seen in Figure 6, compared to the control solution, the αTS-PTX-NLC showed a maximum drug release of about 40%, while the αTS-PTX-PEG-NLC exhibited the release of about 67% in similar time, and the drug concentration reached plateau until the end of the study (48 h). This was confirmed with the obtained *f*_2_ value of 22 and 29 for αTS-PTX-NLC and αTS-PTX-PEG-NLC, respectively. Thus, the drug release from the NLC formulations was not complete, and the drug was withheld within the nanoparticles. The pattern of the drug release can be attributed to the loosely bound and superficial drug in the NLC that detaches quickly, while the drug at the core of NLCs remains intact and is not released. This was confirmed by measuring the particle size and PDI of the nanoparticles in release media at the end of the study. A similar drug release profile was observed in the literature by Olerile et al. They observed a cumulative release of about 40% in 48 h, and the further release of the drug could have been attributed to the higher drug loading of about 4% *w*/*w* in their formulation [21].

The drug that is released will be immediately bioavailable; however, the incomplete release of the drug should not be correlated to 50% bioavailability. It is likely that the drug-loaded NLCs will be internalized by the cells through receptor-mediated endocytosis or phagocytosis. Within the intracellular space, the NLCs become degraded by the cellular enzymes (lipase enzymes), and thus, the drug is released within the cells [56,57,58]. The cellular internalization of NLCs has been demonstrated by in vitro and in vivo methods. Gökçe et al. demonstrated the internalization of cyclosporine A (CsA)-loaded lipid nanoparticles with rabbit corneal endothelial (RCE) cell lines [59]. In previous studies, they have demonstrated the degradation of the lipid nanoparticles to release the CsA [60]. Kakkar et al., Patil et al., Tatke et al., and others have demonstrated higher drug bioavailability in the aqueous humor and vitreous humor from drug-loaded lipid nanoparticles as compared to the free drug solution, indicating higher permeation of the lipid nanoparticles in the ocular tissues [27,61,62].

The drug release profiles obtained from the formulations were further analyzed by applying different mathematical models such as zero-order release, first order release, Higuchi model and Korsmeyer–Peppas model. The goodness of fit (R^2^) for each of the release profiles using the mentioned models was determined, and the model with the highest obtained value for the R^2^ was considered as the optimal mathematical model to describe release kinetics for the formulations.

From Table 8, the zero-order model explains the release of the drug when there is a constant rate irrespective of the concentration, and it is a function only of time [63]. Meanwhile, the release of the drug can be termed as the first order when the rate of the release of the drug is a function of the remaining concentration of the drug [63]. The Higuchi model for the release kinetics states that the rate of the release of the drug is a function of the square root of the time [41,64]. Lastly, Korsmeyer–Peppas is a semi-empirical model that can usually be used to describe the mechanism of the drug release phenomenon consisting of diffusion or swelling [41,65].

Table 8 presents the R^2^ values for each of the models. For the αTS-PTX-NLC with an R^2^ value of 0.9880 and the αTS-PTX-PEG-NLC with the R^2^ values of 0.9991, the Korsmeyer–Peppas models indicated the best fit for the data. For the Korsmeyer–Peppas models, the n value of less than 0.5 indicates Fickian diffusion [66] The n values for the αTS-PTX-NLC and αTS-PTX-PEG-NLC were 0.7408 and 0.7409, respectively, indicating a non-Fickian diffusion behavior for the drug release (when 0.5 < n < 1) [66]. This is supported by the data obtained for the NLC formulations, where a plateau was observed after a certain percentage of drug release from the NLC formulations, and a steady concentration was maintained, indicating no effect of the concentration of drug remaining, thus a non-Fickian diffusion mechanism.

### 3.4. Sterilization of Formulation

**Figure 7:**
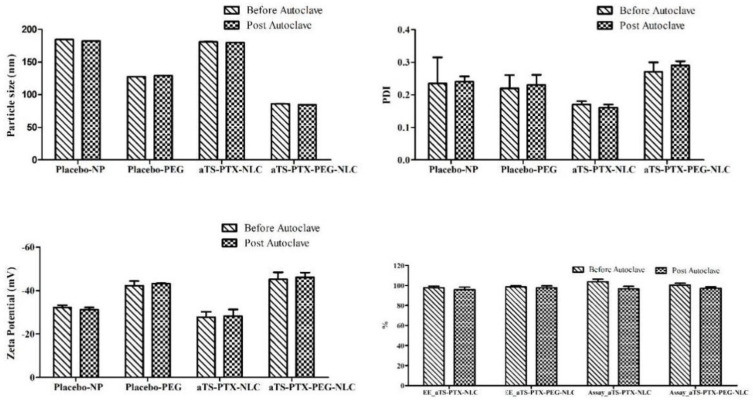
Comparison of the physical and chemical characteristics of the placebo and αTS-PTX-NLC formulation before and after autoclave.

The formulations were autoclaved at a temperature of 121 °C and 15 psi pressure by a 15 min cycle. To understand the effect of sterilization on the physiochemical characteristics of NLC, the particle size, PDI, zeta potential, assay and entrapment efficiency were determined before and after autoclave and were compared and are shown in Figure 7.

There was no significant difference (*p* > 0.05) was found for the particle size, PDI, ZP, assay and entrapment efficiency of the non-autoclaved and autoclaved NLC formulations.

### 3.5. Stability Studies

The stability studies were performed on the autoclaved formulations, both placebo and αTS-PTX-NLC (F5) formulations. The data for the autoclaved samples stored at 4 °C indicated a non-significant (*p* > 0.05) difference between the physiochemical characteristics of the non-autoclaved and autoclaved formulations. The stability of the NLC formulations was determined by measurement of particle size distribution, zeta potential, assay, and entrapment efficiency. During the preliminary, it was found that the NLC formulations were not stable for more than 15 days when stored at 25 °C. The nanoparticles stored at higher temperature (25 °C) exhibited less stability as compared to the NLC stored at 4 °C. This phenomenon can be described by the changes in the micro-viscosity (rigidity of the surfactant film on the NLC), decrease in zeta potential, as well as the increase in the kinetic energy of the system, resulting in higher velocity collisions of the NLCs that can overcome the electrostatic repulsion necessary to prevent agglomerations [67]. The autoclaved formulations showed good stability up to 60 days (last time point measured).

**Figure 8:**
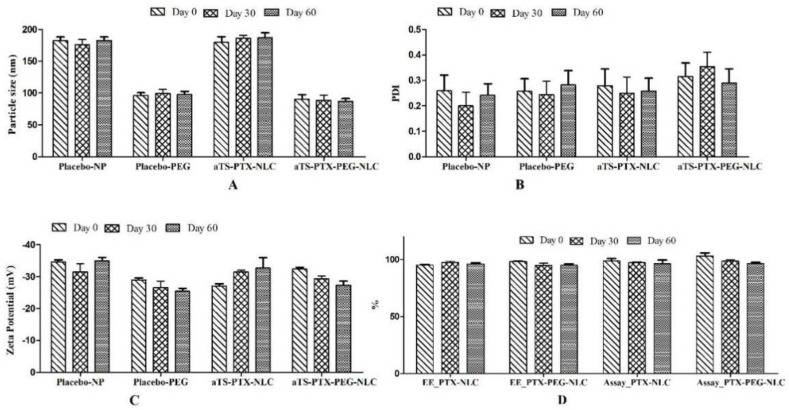
Stability studies for αTS-NLC (F5) formulation stored at 4 °C for up to 60 days (last time point measured). (**A**) Particle size, (**B**) PDI, (**C**) Zeta potential, (**D**) Entrapment Efficiency and Assay.

As illustrated in Figure 8, the placebo as well as αTS-PTX-NLC (F5) formulations showed stability in terms of particle size, PDI, zeta potential, assay, and entrapment efficiency. The PEGylated NLCs exhibited lower difference between Day 1 and Day 60 in case of particle size, PDI and zeta potential. There was a non-significant difference between the assay and entrapment efficiency of the NLC until the last time-point measured.

### 3.6. Electron Microscopy of NLC

**Figure 9:**
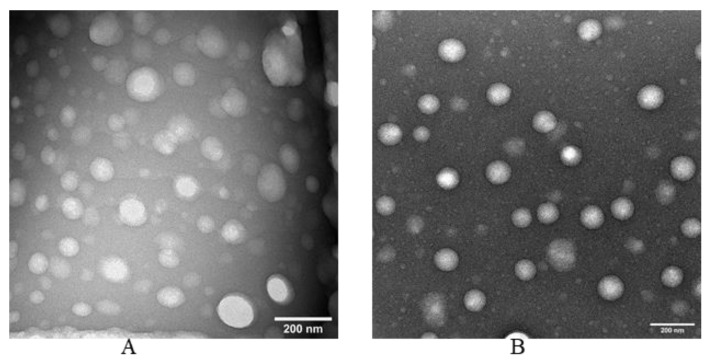
Electron microscopy images of (**A**) αTS-PTX-NLC and (**B**) αTS-PTX-PEG-NLC.

From the TEM images (Figure 9), it can be observed that both the αTS-PTX-NLC and αTS-PTX-PEG-NLC exhibited a spherical shape and a uniform particle size distribution, which is in agreement with the particle size data obtained from DLS measurements.

## 4. Conclusions

This study reports the preparation and optimization of α-TS-based PTX loaded NLC optimized by using repeated measures ANOVA experimental design. To the best of our knowledge, this is the first study that reports the α-TS-based lipid nanoparticles that use α-TS as a solid lipid. The optimized αTS-PTX-NLC and αTS-PTX-NLC were stable on sterilization by autoclaving and had a small particle size, narrow PDI, and high entrapment efficiency and were found to be stable for a minimum of 60 days. In addition, from the drug loading and release behavior of the formulated αTS-PTX-NLC and αTS-PTX-PEG-NLC, as compared to the previously reported formulations, it can be said that these NLC formulations will be effective against the cancerous cell lines.

## Figures and Tables

**Table 1:** Quality Target Product Profile for PTX-NLC.

Attribute	QTPP	Justification
Dosage form type	Lipid nanoparticles	Biocompatible and helps to increase the bioavailability of the drug with controlled release. Enhance cellular uptake of the drug.
Route of administration	Precorneal and intravitreal injection	Administration in the precorneal cul-de-sac is the most patient-friendly route for ocular drug delivery. While the intravitreal injection is a bit challenging, it can deliver a high dose of drug at the site and help achieve drug depot formation.
Storage stability	At least 2 months in suspension form	The NLCs need to be stable for a longer duration in suspension form for multiple administration. The stability on dilution to ease dose adjustment.

**Table 2:** Critical Quality Attributes (CQA) used for the PTX-NLC.

Quality Attributes	Target	Is This a CQA?	Justification
Physical attributes		No	Color, odor, and appearance were not considered as critical as these are not directly linked to patients.
Drug content	More than 95%	Yes	Drug content is vital for any pharmaceutical dosage form for attaining the maximal plasma concentration of a drug. The drug content does not decrease substantially in the selected method and hence was regarded as moderately critical.
Particle size	Less than 200 nm	Yes	As NLC was administered through the ocular route, particle size will have a significant impact on the drug absorption. So, it was considered as critical.
Entrapment efficiency	More than 95%	Yes	Higher entrapment efficiency is vital for accomplishing maximum drug release regulation from dosage form and hence the therapeutic concentration of the drug. Thus, it was considered as critical.
Amount of drug release after 60 min	Less than 50%	Yes	The amount of drug released in the first hour will decide the promptness of the formulation effect; thus, it is taken as critical. The deterred release will help to retain the drug within the NLC.

**Table 3:** Formulation compositions for optimization of the lipid content and lipid ratio.

#	αTS (%)	CP90:αTS	PTX (% *w*/*v*)	Particle Size (nm)		PDI		Zeta Potential (mV)	
				Day 1	Day 30	Day 1	Day 30	Day 1	Day 30
F1	3.00	0.33	0.1	182.83 ± 5.26	232.92 ± 24.30	0.19 ± 0.01	0.27 ± 0.06	−30.51 ± 0.72	−31.16 ± 4.20
F2	3.00	0.5	0.1	166.13 ± 5.53	196.47 ± 5.92	0.13 ± 0.01	0.32 ± 0.15	−29.77 ± 2.62	−30.01 ± 4.80
F3	3.00	0.67	0.1	153.97 ± 3.95	244.81 ± 8.21	0.16 ± 0.04	0.59 ± 0.31	−29.08 ± 2.65	−31.42 ± 0.98
F4	4.00	0.33	0.1	208.97 ± 10.52	186.16 ± 6.79	0.20 ± 0.01	0.19 ± 0.05	−31.25 ± 2.41	−28.85 ± 4.14
F5	4.00	0.5	0.1	214.17 ± 11.34	177.51 ± 5.16	0.12 ± 0.08	0.19 ± 0.03	−30.54 ± 3.28	−31.22 ± 2.34
F6	4.00	0.67	0.1	195.77 ± 3.67	175.28 ± 6.15	0.17 ± 0.00	0.18 ± 0.04	−29.11 ± 2.72	−30.31 ± 2.26
F7	5.00	0.33	0.1	237.93 ± 9.25	214.21 ± 6.93	0.16 ± 0.03	0.21 ± 0.04	−31.18 ± 3.42	−30.83 ± 1.48
F8	5.00	0.5	0.1	223.30 ± 8.95	199.92 ± 5.34	0.19 ± 0.02	0.17 ± 0.04	−29.47 ± 0.77	−29.42 ± 2.67
F9	5.00	0.67	0.1	198.50 ± 1.47	203.66 ± 6.62	0.17 ± 0.02	0.17 ± 0.05	−28.65 ± 2.67	−29.89 ± 1.97

**Table 4:** Within-subject effects for repeated measures ANOVA analysis.

Within-Subject Effects	*p*-Value		
	Particle Size	PDI	Zeta Potential
Stability	0.001	<0.001	0.288
Stability*αTS	<0.001	<0.001	0.378
Stability*CP90:αTS	<0.001	0.101	0.057
Stability*αTS*CP90:αTS	<0.001	0.029	0.566

* Denotes the interaction effect of the independent variables.

**Table 5:** Between-subject effects for repeated measures ANOVA analysis.

Between-Subject Effects	*p*-Value		
	Particle Size	PDI	Zeta Potential
αTS	<0.001	0.009	0.941
CP90:αTS	0.001	0.275	0.772
αTS*CP90:αTS	0.02	0.233	0.938

* Denotes the interaction effect of the independent variables.

**Table 6:** Formulations for optimization of DSPE-PEG-2k.

αTS (% *w*/*v*)	CP90 (% *w*/*v*)	PTX (% *w*/*v*)	DSPE-PEG-2k (% *w*/*v*)	Particle Size (nm)	PDI
4.0	2.0	-	0	183.30 ± 8.41	0.19 ± 0.03
4.0	2.0	-	0.5	103.73 ± 1.79	0.18 ± 0.04
4.0	2.0	-	1	72.74 ± 0.55	0.21 ± 0.04
4.0	2.0	-	1.5	123.70 ± 8.62	0.22 ± 0.01
4.0	2.0	0.1	0	218.83 ± 6.38	0.09 ± 0.05
4.0	2.0	0.1	0.5	91.80 ± 1.69	0.20 ± 0.00
4.0	2.0	0.1	1	82.37 ± 1.61	0.28 ± 0.05
4.0	2.0	0.1	1.5	92.40 ± 4.14	0.37 ± 0.08

**Table 7:** ANOVA table for the effect of the DSPE-PEG-2k and drug on the particle size and PDI of NLC formulations.

Variable	*p*-Value	
	Particle Size	PDI
DPPE-PEG-2k	<0.001	<0.001
PTX	0.821	0.058
DSPE-PEG-2K*PTX	<0.001	0.002

* Denotes the interaction effect of the independent variables.

**Table 8:** Mathematical fitting (goodness of fit values (R^2^)) of the drug release of PTX from αTS-PTX-NLC and αTS-PTX-PEG-NLC formulations.

	Zero Order	First Order	Higuchi Model	Korsmeyer–Peppas Model	
	R^2^				n
αTS-PTX-NLC	0.9376	0.9824	0.7445	0.9880	0.7408
αTS-PTX-PEG-NLC	0.9102	0.9744	0.9793	0.9991	0.7409

## Data Availability

The data presented in this study are available upon request from the corresponding author.
